# Implant survival and factors associated with failure of cemented custom-made distal femoral megaprostheses after tumor resection

**DOI:** 10.1051/sicotj/2026020

**Published:** 2026-04-29

**Authors:** Vasileios Apostolopoulos, Michal Mahdal, Marián Kubíček, Lukáš Pazourek, Petr Boháč, Luboš Nachtnebl, Tomáš Tomáš

**Affiliations:** 1 First Department of Orthopaedic Surgery, St. Anne’s University Hospital and Faculty of Medicine, Masaryk University Brno Czechia; 2 Institute of Solid Mechanics, Mechatronics and Biomechanics, Faculty of Mechanical Engineering, Brno University of Technology Brno Czechia

**Keywords:** Distal femur replacement, Megaprosthesis, Bone tumor, Implant survival, Mechanical failure

## Abstract

*Background*: Distal femoral megaprosthetic reconstruction is a standard limb-salvage procedure after tumor resection. This study aimed to evaluate implant survival and associated factors, the incidence of mechanical failure, and functional outcomes following reconstruction with cemented custom-made distal femoral megaprostheses. *Methods*: Fifty-seven patients who underwent distal femoral tumor resection followed by reconstruction with a cemented custom-made distal femoral megaprosthesis between 2010 and 2024 were retrospectively analyzed. Implant survival was evaluated using Kaplan–Meier analysis, and associations with outcomes were assessed using Cox proportional hazards and Fine–Gray competing-risk regression models. The analyzed risk factors included age, sex, resection length, stem diameter, fixation length, and functional score. Functional outcomes were assessed using the Musculoskeletal Tumor Society (MSTS) score. *Results*: Implant survival remained above 60% at the latest follow-up, with survival rates of 100% at 12 months, 93.5% at 24 months, and 72.9% at 60 months. No clinical or implant-related geometric variables were significantly associated with implant survival. The cumulative incidence of mechanical failure was 7% at 48 months and approximately 15% at the latest follow-up, with no association between mechanical failure and resection length, stem diameter, or fixation length. Functional outcomes were favorable, with a mean MSTS score of 21.6 ± 3.9. *Conclusion*: Cemented custom-made distal femoral megaprostheses demonstrated satisfactory mid- to long-term survival following tumor resection. In this cohort, none of the evaluated variables were significantly associated with implant survival. The incidence of mechanical failure remained relatively low, and geometric implant parameters were not significantly associated with mechanical failure. Functional outcomes were favorable, with most patients achieving good or excellent MSTS scores.

## Introduction

Distal femoral tumor resection followed by endoprosthetic reconstruction is a mainstay of limb-salvage surgery for primary and metastatic bone tumors of the knee, providing immediate structural stability and facilitating early mobilization [1–3]. Despite advancements in implant design, overall complication rates remain substantial, and implant failure continues to be a significant clinical challenge. Implant failures are commonly categorized by mechanism, with mechanical modes – such as aseptic loosening and structural breakage – representing a relevant subset that may reflect implant design and biomechanical factors [4–6].

Modern practice increasingly favors uncemented fixation techniques because of their potential for biological integration [7]. Cemented custom-made tumor knee endoprostheses are still widely used, particularly in patients with poor bone quality or extensive resection defects, as they provide immediate fixation and mechanical stability [7–9]. However, evidence regarding long-term survival, functional outcomes, and specific mechanical failure patterns in cemented systems remains limited [10–14]. Many existing reports in the literature combine heterogeneous implant types and fixation methods, which obscures the influence of distinct mechanical variables on outcomes [7, 10, 15–17].

Although distal femoral replacement is a well-established reconstructive option after tumor resection, risk factors for implant survival and the influence of geometric implant parameters on outcomes remain insufficiently defined. In particular, the roles of resection length, stem diameter, and fixation length are difficult to assess from the existing literature. By evaluating these parameters in a homogeneous cohort of patients treated with cemented custom-made distal femoral replacements, the present study provides more focused data on implant survival, mechanical failure, and functional outcome in this specific reconstructive setting. The primary aim of this study was to evaluate implant survival and factors associated with implant survival following distal femoral tumor resection reconstructed with cemented custom-made distal femoral megaprostheses. The secondary aim was to assess the incidence and predictors of mechanical failure. A further aim was to describe functional outcomes in this cohort.

## Methods

### Study design and patients

This retrospective cohort study included 57 patients who underwent distal femoral tumor resection followed by reconstruction with a cemented custom-made distal femoral megaprosthesis (Beznoska) between 2010 and 2024 at a single tertiary referral center. Indications for surgery included primary and metastatic bone tumors of the distal femur. Demographic and tumor characteristics are summarized in Table 1.

**Table 1:** Demographic data of patients who underwent tumor resection followed by reconstruction with a cemented custom-made distal femoral megaprosthesis, Beznoska, between 2010 and 2024. Continuous variables are presented as mean ± SD. Categorical variables are presented as number with percentages.

Features	Overall
Number of patients (n)	57
Age at inclusion (years)	57.5 ± 16.7
Sex (*n*, %)	
Female	33 (57.9%)
Male	24 (42.1%)
Follow-up (months), median (IQR)	45.0 (20.0–91.0)
Defect length (cm)	13.5 (±4.9)
Stem diameter (mm)	15.1 (±1.4)
Length of fixation (cm)	17.9 (±4.5)
Bone tumor (*n*, %)	
High-grade chondrosarcoma	6 (10.5%)
High-grade osteosarcoma	13 (22.8%)
Other sarcoma types	6 (10.5%)
High-grade lymphoma	2 (3.5%)
Giant cell tumor of bone	7 (12.3%)
Metastatic tumor	23 (40.4%)

### Variables and outcome measures

The primary outcome was implant survival, defined as the time from primary implantation to prosthesis failure for any cause. Implant failure was defined as revision or removal of any prosthetic component. Mechanical failure was defined according to the Henderson classification as type 2 (aseptic loosening) or type 3 (structural failure) [4, 5]. Mechanical variables of interest included length of defect, stem diameter, and fixation length. Length of defect was defined as the distance from the distal femoral joint line to the proximal osteotomy level. Stem diameter was defined as the outer diameter of the femoral intramedullary stem of the megaprosthesis as documented in the implant specification. Length of fixation was defined as the intramedullary length of the femoral stem within the remaining host bone. Measurements of fixation length and defect length were obtained from operative records and postoperative imaging. The femoral stem was implanted using polymethylmethacrylate bone cement with standard cementing technique, including vacuum mixing, pulsatile lavage, retrograde cement insertion, and pressurization. Functional outcome was assessed using the Musculoskeletal Tumor Society (MSTS) [18] score when available. Follow-up assessments were conducted monthly during the first 3 months, every 3 months for the subsequent 2 years, every 6 months until year five, and annually thereafter.

### Statistical analysis

Statistical analysis was performed using R (RStudio). Continuous variables are presented as mean ± standard deviation or median (interquartile range), and categorical variables as counts and percentages. Normality was assessed using the Shapiro–Wilk test and graphical methods. A *p*-value < 0.05 was considered statistically significant. Reverse Kaplan–Meier was used to calculate the median follow-up time. Implant survival was analyzed using the Kaplan–Meier analysis. Associations with outcomes were assessed using Cox proportional hazards regression, with proportionality tested by Schoenfeld residuals. Mechanical failure was additionally analyzed using cumulative incidence functions and competing-risk regression with the Fine–Gray model.

## Results

### Implant survival and associated factors

**Figure 1:**
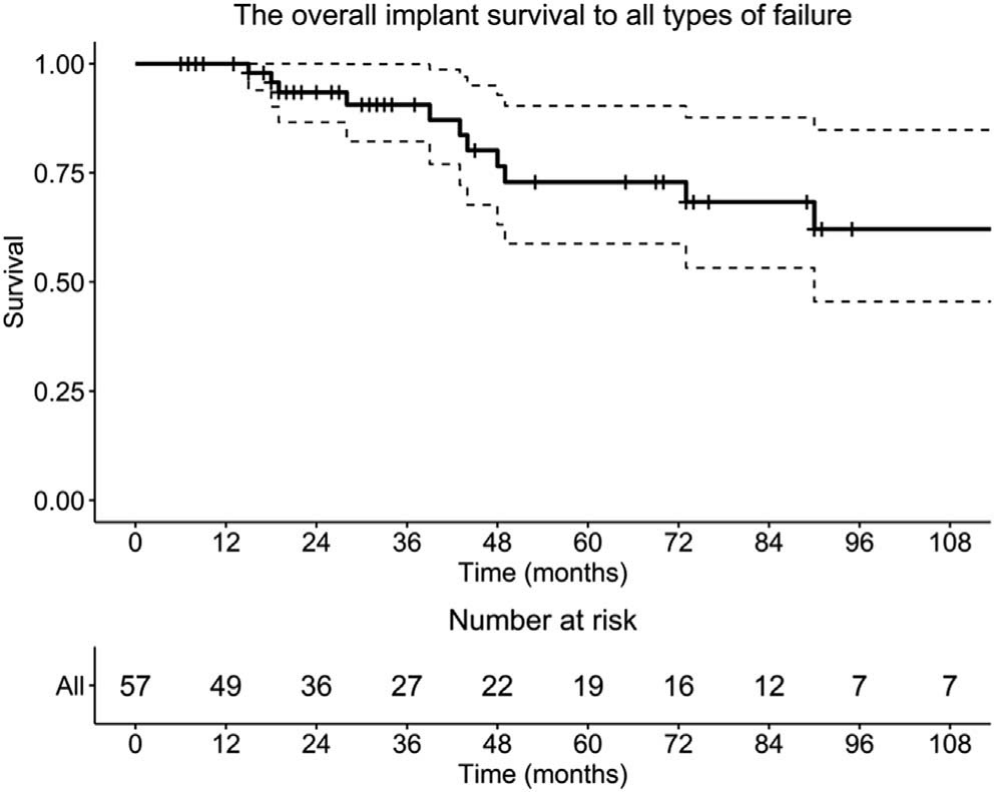
Kaplan–Meier curve showing overall implant survival following distal femoral tumor resection, reconstructed with a cemented custom-made distal femoral megaprosthesis.

**Figure 2:**
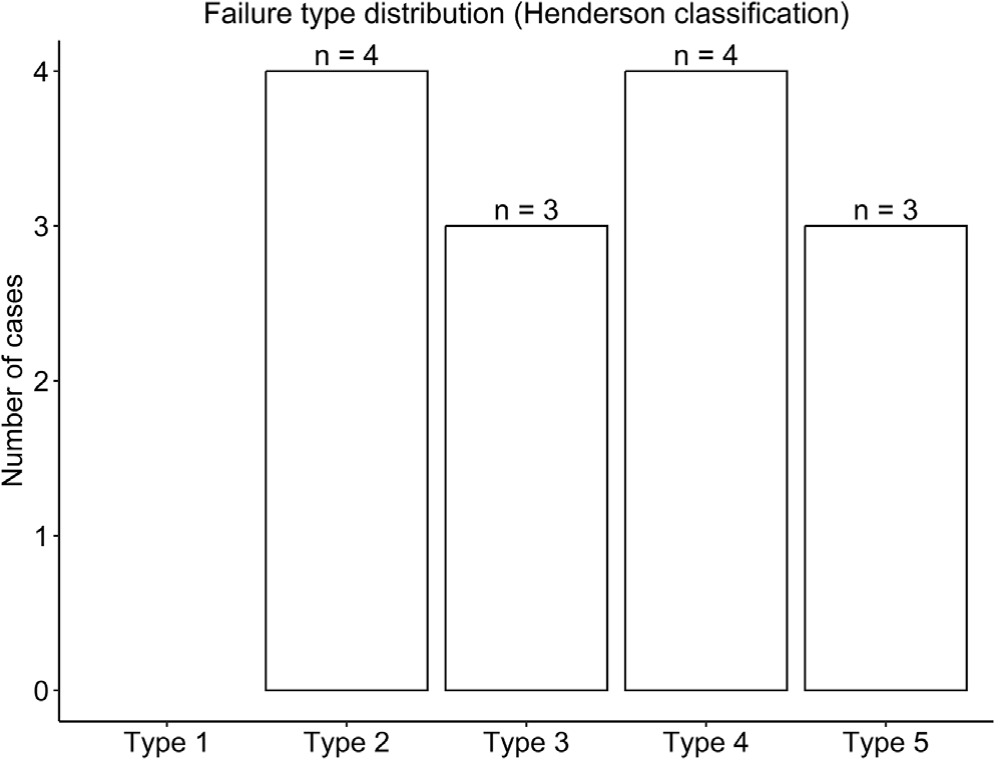
Distribution of implant failure types according to the Henderson classification in patients treated with cemented custom-made distal femoral megaprostheses.

Overall implant survival remained above 60% at the latest follow-up, with no clinical or mechanical variables significantly associated with implant survival. Implant survival was 100% at 12 months, 93.5% at 24 months, and 72.9% at 60 months (5 years). A further decline was observed beyond 5 years, with survival remaining above 60% at the latest follow-up. Most failures occurred between 24 and 60 months postoperatively (Figure 1). Failure type distribution is shown in Figure 2.

Univariable Cox regression analysis did not identify any statistically significant predictors of overall implant survival (Table 2). Neither age, length of defect, stem diameter, fixation length, MSTS score, nor sex was associated with an increased risk of implant failure (all *p* > 0.05).

**Table 2:** Cox proportional hazards analysis of factors associated with overall implant survival.

Outcome	Variable	HR (95% CI)	*p*-Value
Overall implant survival	Age	1.009 (0.975–1.045)	0.595
	Length of defect	1.005 (0.994–1.017)	0.382
	Stem diameter	1.046 (0.728–1.503)	0.809
	Length of fixation	1.004 (0.991–1.016)	0.556
	MSTS (*N* = 40)	0.881 (0.718–1.080)	0.223
	Sex: Male	0.743 (0.224–2.459)	0.626

### Mechanical failure incidence and associated factors

**Figure 3:**
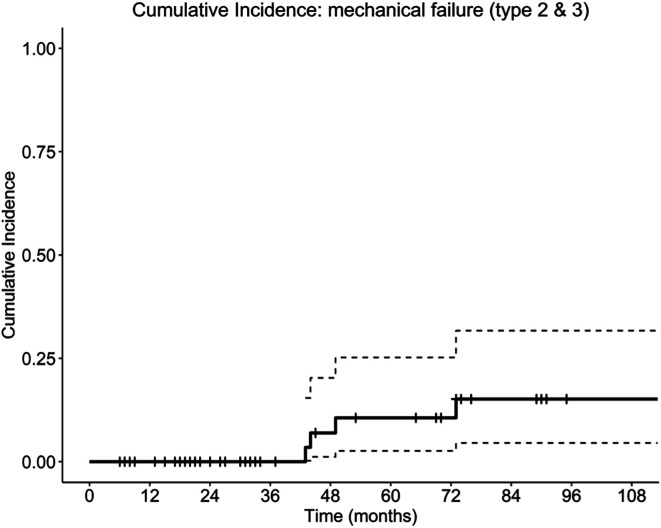
Cumulative incidence of mechanical failure (Henderson types 2 and 3) following distal femoral tumor resection reconstructed with a cemented custom-made distal femoral megaprosthesis.

Mechanical failure occurred at a relatively low incidence and was not significantly associated with the evaluated geometric implant parameters. The cumulative incidence of mechanical failure (Henderson types 2 and 3) remained low during the early postoperative period, with no mechanical failures observed within the first 36 months. The cumulative incidence of mechanical failure was 7% at 48 months and increased to 11% at 60 months, reaching approximately 15% at the latest follow-up (Figure 3).

**Figure 4:**
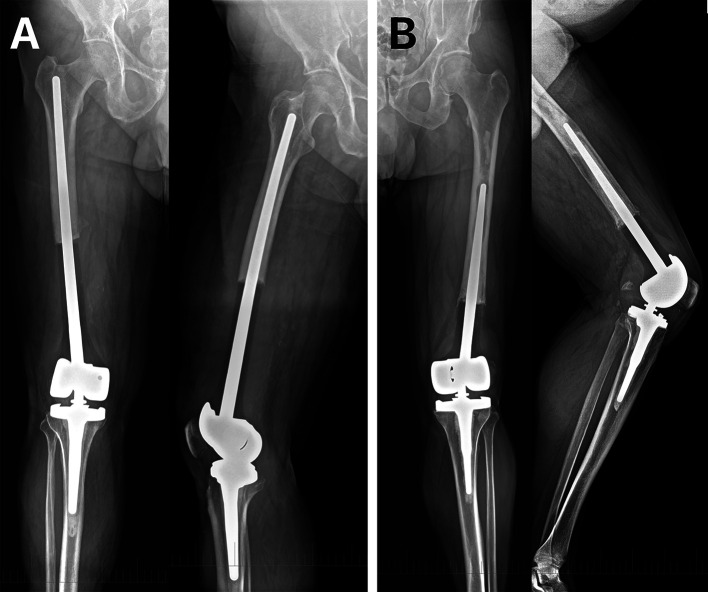
Representative radiographs of cemented custom-made distal femoral megaprosthetic reconstruction after tumor resection. (A) Patient following resection of a conventional grade 2 chondrosarcoma with a 20 cm distal femoral defect reconstructed using a cemented custom-made distal femoral replacement (stem diameter 14 mm, fixation length 21 cm). (B) Patient with malignant epithelioid hemangioendothelioma treated with distal femoral resection of 14 cm and reconstruction using a cemented custom-made megaprosthesis (stem diameter 16 mm, fixation length 17 cm), demonstrating aseptic loosening 6 years after implantation.

In competing-risk regression analysis using the Fine–Gray model, no mechanical variable was statistically significantly associated with the risk of mechanical failure (Table 3). Length of defect, stem diameter, and fixation length showed no statistically significant association with mechanical failure (all *p* > 0.05). Representative radiographs of mechanical failure due to aseptic loosening of a cemented custom-made distal femoral megaprosthesis are shown in Figure 4.

**Table 3:** Competing-risk regression analysis (Fine–Gray model) of factors associated with mechanical failures.

Outcome	Variable	HR (95% CI)	*p*-value
Implant survival to mechanical failure	Length of defect	0.995 (0.981–1.009)	0.500
	Stem diameter	0.879 (0.548–1.409)	0.590
	Length of fixation	0.998 (0.964–1.033)	0.910

### Functional outcomes

Functional outcomes were generally favorable, with most patients achieving good or excellent MSTS scores. Functional outcome assessed using the MSTS score (available in 40 patients) showed a mean score of 21.6 ± 3.9. According to categorical MSTS grading, 45.0% of patients achieved excellent results (76–100%), 47.5% had good outcomes (51–75%), and 7.5% had fair outcomes (26–50%), with no patients classified as having poor (0–25%) functional results.

## Discussion

Distal femoral megaprosthetic reconstruction is a widely used limb-salvage procedure following tumor resection, but implant survival and the influence of geometric implant parameters remain incompletely understood. In this study of patients treated with cemented custom-made distal femoral megaprostheses, implant survival remained above 60% at the latest follow-up, mechanical failure occurred at a relatively low incidence, and none of the evaluated geometric parameters – resection length, stem diameter, or fixation length – were significantly associated with implant survival or mechanical failure. Functional outcomes were generally favorable, with most patients achieving good or excellent MSTS scores.

Several limitations should be acknowledged. First, the retrospective design inherently carries the risk of selection and information bias. Second, although the cohort represents a relatively homogeneous group of patients, the sample size remains limited, which may reduce the statistical power to detect subtle associations between mechanical parameters and implant survival. Finally, this study included only cemented custom-made distal femoral megaprostheses from a single institution and manufacturer, which may limit the generalizability of the findings to other implant systems or fixation techniques.

Implant survival in the present cohort was comparable to previously reported outcomes for distal femoral tumor endoprostheses [3, 15, 19–25]. Bergin et al. reported long-term implant survival rates of 73.3% at 10 years and 62.8% at 15 years in patients treated with distal femoral endoprosthetic reconstruction [11]. In a systematic review by Haijie et al., the mean 5-year implant survival rate was 78.3%, compared with 72.9% in our series [19]. Moreover, implant survival remained above 60% at the latest follow-up, supporting the durability of cemented fixation even beyond 5 years in this complex oncologic population. Several studies have identified multiple factors associated with implant survival following distal femoral reconstruction, including patient-related variables, tumor characteristics, and implant design features [3, 15, 19–21, 26]. However, in the present study, none of the evaluated variables – including age, sex, resection length, stem diameter, fixation length, or functional score – were significantly associated with implant survival.

In the present study, the cumulative incidence of mechanical failure remained relatively low, reaching 7% at 48 months and approximately 15% at the latest follow-up. None of the analyzed geometric implant parameters – resection length, stem diameter, or fixation length – were significantly associated with mechanical failure. Reported rates of mechanical failure after distal femoral megaprosthetic reconstruction vary across cohorts but are generally within a comparable range, with mid-term incidences of approximately 5–6% in some series and 5-year implant failure rates around 12–17% in others [10, 11, 27]. Although prior studies report no significant difference between cemented and cementless fixation [19], the delayed onset of mechanical failure in our cohort suggests that cemented fixation provides reliable primary stability, which may be advantageous in patients requiring immediate weight bearing or receiving adjuvant therapy. The lack of an association between resection length and implant survival is noteworthy, as greater resection length has been identified as a major risk factor for aseptic loosening in prior studies [28, 29]. This correlation is typically attributed to increased bending moments and higher stresses at the stem–cement interface in larger resections [11]. Similarly, stem diameter and fixation length, which are commonly considered critical determinants of load distribution and resistance to loosening [19, 28, 29], did not emerge as significant predictors in the present study. The results indicate that mechanical failure in cemented distal femoral megaprostheses is influenced by multiple interacting factors and cannot be determined by simple geometric parameters alone.

Functional outcomes were encouraging, with over 90% of patients achieving good or excellent results according to categorical MSTS grading. The mean MSTS at the most recent follow-up was 21.6 points, which is comparable to previous studies [21, 30]. These findings highlight the ability of distal femoral megaprostheses to restore acceptable limb function and quality of life following extensive tumor resection. Satisfactory functional outcomes were maintained despite substantial defect length, suggesting that the mechanical reconstruction was sufficient to support daily activities. The main findings of selected cohort studies on distal femoral replacement after tumor resection are summarized in Table 4.

**Table 4:** Summary of key published cohort studies on cemented distal femoral megaprosthetic reconstruction after tumor resection.

Study	*n*	Type of implant	Fixation	Follow-up (mean)	5-year implant survival	Mechanical failure	Functional outcome (MSTS)
Schwartz et al. 2010 [12]	186	Custom–made + modular	Cemented	96 months	83.5%	18.8%	Not reported
Piakong et al. 2020 [14]	195	Modular	Cemented	78 months	74%	7.2% (aseptic loosening)	Not reported
Bergin et al. 2011 [11]	104	Modular	Cemented	5.6 years	76.1%	10.7%	Not reported
Houdek et al. 2016 [3]	152	Custom–made + modular	Cemented	10 years	76%	21.1%	25
Holm et al. 2025 [10]	59	Modular	Cemented	3 years	88%	13%	17
Sharma et al. 2006 [24]	77	Modular	Cemented	52 months	84%	0% (aseptic loosening)	30
Ahlmann et al. 2006 [22]	78	Modular	Cemented	37 months	75%	15.2%	22.9
Mattei et al. 2020 [25]	136	Modular	Cemented	81 months	78%	18%	24.6
Jeys et al. 2008 [23]	228	Modular	Cemented	9 years	68.6% (at 10 years)	21.1%	Not reported
Present study	57	Custom–made	Cemented	49 months	72.9%	15%	21.6

## Conclusion

Cemented custom-made distal femoral megaprostheses demonstrated satisfactory mid- to long-term implant survival following tumor resection. In this cohort, none of the evaluated variables were significantly associated with implant survival. The incidence of mechanical failure remained relatively low, and geometric implant parameters were not significantly associated with mechanical failure. Functional outcomes were favorable, with most patients achieving good or excellent MSTS scores. These findings suggest that failure after cemented distal femoral reconstruction is multifactorial and not solely determined by basic geometric implant characteristics.

## Data Availability

The data that support the findings of this study are available from the corresponding author upon reasonable request.
